# Offering and Asking for Help with Domestic Chores in Couple Relationships

**DOI:** 10.3390/ijerph20043708

**Published:** 2023-02-19

**Authors:** Marius Marici, Otilia Clipa, Maria-Doina Schipor, Remus Runcan, Ana-Maria Andrei

**Affiliations:** 1 Faculty of Education Sciences, Ștefan cel Mare University, 720229 Suceava, Romania; 2 Faculty of Educational Sciences, Psychology and Social Work, Aurel Vlaicu University of Arad, 310032 Arad, Romania; 3 Psychology and Educational Sciences Faculty, Alexandru Ioan Cuza University, 700506 Iași, Romania

**Keywords:** offering help, asking for help, gender differences, couple relationships, domestic chores, intuitive vs. verbal communication

## Abstract

Domestic chores are a topic of great interest for couple relationships since they are a source of conflict between intimate partners. The purpose of the present research is to investigate offering and asking for help with domestic chores and the respondents’ tendency to be intuitive or verbal or to do chores alone. A vignette applied to children and married adults. The respondents were 116 boys and 116 girls and 110 male partners and 300 female partners, who completed individual questionnaires, online using Google Forms, regarding helping behaviour. Research findings indicate that men are more verbal and women are more intuitive when offering help, but when asking for help with domestic chores, men and women are statistically similar. The present research raises questions about the role of gender differences in couple relationships, and about educational solutions for couples and provides opportunities for future research.

## 1. Introduction

Helping is a universal practice [1] and it is a key component in couple relationships since disagreement over the sharing of chores is a reason for relationship breakdowns [2]. Helping or seeking to help fall into the broad category of prosocial behaviours or the narrower category of altruism, and people can manifest different styles of helping. The literature has drawn a distinction between helping behaviours which can be direct (verbally expressing the specific help needed or offered) or indirect (still verbal, but without mentioning the specific help needed or offered) [3]. The scientific literature dealt more with verbal-based helping. As we searched the scientific databases we could not find any articles about offering or waiting for help without words, which led us to the idea that this could be a new category of helping behaviour, characteristic of couple relationships. The present study refers to intuitive help as help offered or received without any verbal communication.

The idea for the present study started from observations with psychotherapy clients in private practice (of Marius Marici). Dozens of couples were found to complain about household chores. In the following dialogue, the female partner demands help without specifically telling her male partner what to do, while the male partner complains about not understanding what he has to do since he is not being told:

*Female partner*: You don’t help me when I need it.

*Male partner*: I don’t know when you need me to do something.

*Female partner*: You must be responsible and see that I need help and do things without me telling you to do so.

*Male partner*: I do everything you ask me to do. When did you ask me something and I didn’t do it? Everything you asked me I did.

Based on the notes kept during psychotherapy sessions, 57 of the 82 cases that were handled stated that household chore misunderstandings start from the fact that women expect men to notice what needs to be completed and do chores without specifically being told what to do; men, on the other hand, complain they are only willing to do chores when they are told what needs to be accomplished. Otherwise, they are unable to comprehend their partner’s expectations.

As a result, the present study focuses on judgement differences between males and females in an imaginary couple situation, based on their choice of asking for or offering help by being intuitive or verbal. Doing the chores alone was also considered.

On the one hand, due to cultural pressures to standardise behaviours in accordance with its norms, including helping behaviour, children, and adults may evaluate the relationship situation similarly. On the other hand, children and adults might judge couple situations differently since there is a gap between experiencing life as a couple and ‘witnessing’ or ‘anticipating’ as children do. Thus, the present research focuses both on children and married respondents in order to better understand the similarities and differences between them.

A search in the literature indicates that there are few articles (if any) that deal with the present topic and studies to investigate this topic in couple relationships are even scarcer.

## 2. Males and Females in Intuitive and Verbal Interactions

Research shows that men and women show differences over a wide range of behaviours [4]. According to the Dual process theory, individuals process information using two independent cognitive systems: System 1 (implicit) and System 2 (explicit), both of which contribute to judgement and decision-making [5]. System 1 is automatic, effortless, fast, relatively undemanding of cognitive capacity, tacit, experiential, and intuitive, based on recognition-primed decisions and implicit inferences or cognitions that are preconscious, based on emotions and rely more on generalisations rather on logic and verbal principles [6,7]. System 2 is analytic, effortful, slow, demanding of cognitive capacity, based on rational choice strategy, explicit, conscious, based on rationality, intentional, and correlates more with verbal expression [5]. System 1 corresponds to intuitive interaction, while System 2 corresponds to verbal interaction.

A study on Romanian participants indicates that students perceive women to be significantly more intuitive than men [8]. Stereotypes about intuition do exist but are highly domain-specific, rather than general [9]. Beyond stereotypes, studies show that women score higher than men on nonverbal decoding ability on the Profile of Nonverbal Sensitivity test [10,11], and ‘women are more polite in the nonverbal aspects of their social interactions than are men. They are guarded in reading those cues that senders might be trying to hide [12] (p. 283). Females score higher than males on intuitive style. Women are considered to be more skilful encoders and decoders of non-verbal communication than men and this serves as an argument for female intuition [13]. Other studies in communication indicate that females tend to soften their demands and statements and males tend to be more direct [14].

Other cues as well as verbal cues, such as facial or behavioural ones, might be used to guide intuition. Research shows that men and boys express fewer affective facial reactions than girls and women [15]. Men are slower and less accurate in their facial expression recognition than women, and they also look less at the eyes. A hypothesis for why females have an advantage in facial expression recognition is that they pay more attention to the eyes than men do [16]. Females would benefit from this mechanism because they are better at being intuitive (not using words).

Frederick’s studies [17] found that men scored higher than women on the Cognitive Reflection Test (measuring intuitive thinking), while [18] did not find such differences. In a review of the literature on female intuition, ref. [19] concluded that two out of five studies supported the stereotype of female intuition. The few studies available on the present subject indicate that the evidence for gender differences in intuition is mixed. Thus, we established two hypotheses: (Hypothesis 1) ‘There will be differences between boys and girls regarding being intuitive, verbal or doing chores alone, in the condition of asking for and offering help.’ (Hypothesis 2) ‘There will be differences between male partners and female partners regarding being intuitive, verbal or doing chores alone, in the condition of asking for and offering help.’

## 3. Gender and Helping Behaviour in Couple Relationships

There are scarce studies that investigate the perceptions of children and adults regarding specific similarities and differences with reference to domestic chores. Few studies, if any, investigated helping behaviour, and compared offering and asking for help, in children and adults, in an imaginary marriage situation, involving domestic chores. Yet, there is a study by Holmberg [20] in which the author interviewed 10 couples who proved their fondness for their partner by taking over their partner’s chores without being asked to help. Thus, this qualitative study suggests that men receive help without being asked verbally at all. The majority of research has concentrated on adults rather than children, on asking for help rather than providing it, and on circumstances other than helping in marriage [3]. Therefore, to our knowledge, this is the first study to compare intuitive versus verbal strategies to initiate helping behaviours in children and adults, regarding domestic chores.

A meta-analysis indicated that males seek help less frequently than females, regardless of age, ethnicity, or nationality [21]. This could be because men might interpret help-seeking as a weakness [21]. However, in romantic relationships, men confessed to loving first more than women [22], a gesture that might be accompanied by more helping behaviour. According to John Gray [23], men are more selfish than women, and when men love their selfishness changes to greater altruism, while women, who are typically more altruistic, when they love they learn to offer more but also they learn to receive more. Love produces changes in their judgement and behaviour. In Gray’s book, we read the following: ‘As a man matures he also learns that he may be giving up himself, but his major change is becoming more aware of how he can succeed in giving. Likewise, as a woman matures she also learns new strategies for giving, but her major change tends to be learning to set limits in order to receive what she wants.’ (p. 34) or ‘Martians have a win/lose philosophy—I want to win, and I don’t care if you lose… Giving primarily to themselves was no longer as satisfying. Being in love, they wanted the Venusians to win as much as themselves.’ (p. 32) and finally ‘On Venus, they lived by a lose/win philosophy—‘I lose so that you can win.’ But after doing this for centuries the Venusians were tired of always caring about one another and sharing everything. They also were ready for a win/win philosophy.’ (p. 34). Although there is little research on the subject, to confirm or inform the statements, the quotes above suggest that judgement in love-based situations is different from judgement in other types of situations.

In addition, in couple relationships, helping behaviour could be interpreted as a sign of the desire to initiate or maintain a relationship [24], and spouses demonstrate love by helping each other [20]. However, helping depends on the perception of how close the relationships are. While a neighbour’s offer to help with gardening would be welcomed, a stranger’s offer would raise suspicion and make us wonder if that person wanted something from us [25]. Since marriage entails a close relationship, both parties are expected to be considerate of one another’s needs and be reciprocal in their helping behaviours. If this is not the case, it may indicate that the relationship is not close or that there is mistrust in the relationship [26]. Becker and Eagly [27] suggest that gender differences in helping depend also on the type of helping that is required. For example, men will be more likely to help in situations where physical force is required, as compared to women, who are more likely to help in situations where nurturance, caring, or helping families is required [28]. Women spend more time on domestic chores than men [29].

Cultural factors might also shape involvement in domestic chores. Women are nowadays as educated as men, and the gender gap regarding domestic chores seems to be narrowing [30]. In the days when their male partners were more stressed with their work, women adjusted and were more involved in house chores, while when women are stressed with their work men are more inclined not to help with it [31]. It is no surprise that women complain more often than men about a lack of household help [32].

Science advances several explanations for gender differences in men and women, including genetic, cultural, and educational. John Gray’s book Men Are from Mars, Women Are from Venus [23] is often cited for focusing on the genetic differences between men and women. Yet, critics bring evidence that even if differences do exist they are small in effect and men and women are more similar than different. The gender similarities hypothesis claims that men and women are similar on most, but not all psychological variables [33]. Culture and socialisation might play an important role in shaping children’s judgement about couple situations, as they see models of couple interactions in their families, in the media, and real-life situations. In addition, the present research refers to an imagined love-based situation, which might activate children’s memories about what they have socially learned in their families about living as a couple [34,35,36,37,38].

Thus, based on the indices found in the research literature, we have established a further two hypotheses: (Hypothesis 3) ‘When offering or asking for help, girls will be similar to female partners in being intuitive, verbal, or doing the chores alone.’, (Hypothesis 4) ‘When offering or asking for help, boys will be similar to male partners in being intuitive, verbal or doing the chores alone.’

## 4. Offering and Asking for Help

There is scarce research on offering and asking for help in couple-related circumstances. The literature highlights mostly the gender imbalance in the distribution of housework. For example, a common separation proposed is between regular duties, usually carried out by women, and episodic tasks (e.g., home repairs), which are usually associated with men [39]. Despite the increased gender equality considered in recent years, gender domestic roles are still unequal [40,41]. Another study showed that men were more likely to view domestic chores as a shared responsibility, while women were more likely to view them as a gendered responsibility. In terms of gender identity, women consider domestic chores as being central to their identity, as a source of power, and are not open to sharing them [42].

Boys are typically less likely than girls to seek help because of masculinity constraints [43]. A study found that ‘boys were less likely than girls both to seek and to offer help’ [44] (p. 220). Men are less likely to seek help as this would be associated with social stigma and would be a sign of weakness affecting their masculinity [45]. Another study did not find any differences between older males and females regarding asking for help when it comes to learning new technologies [46].

Some studies suggest that asking for help is rather domain specific. Girls are more likely to ask for help in a violent situation [47]. A study found that ‘Females consistently suggested at a higher rate than males to seek help from a psychologist than are males.’ [48] (p. 19). ‘Males were the least likely to seek help’ in mental health situations [49] (p. 297). Another study found that ‘men are more involved in traditionally masculine household chores (i.e., home repairs and family management), and women are more involved in traditionally feminine chores (i.e., childcare or shopping)’ [32] (p. 7).

One important question here is whether offering and asking for help in couple relationships, which is a specific domain, are perceived differently by females and males. We presume that asking for and helping in a couple’s life are interpreted as love messages sent between lovers. Thus, offering help might convey the meaning that ‘I love you’ while asking for help might convey the meaning ‘You love me’. Even more, it is very likely that offering help might be more socially acceptable for men as this indicates power and competence, especially in relationships with women.

Thus, as there are some indices that offering and asking for help might trigger different perceptions in males and females we have formulated the following hypotheses: (Hypothesis 5) ‘Offering and asking for help will be different for boys and girls, in verbal, intuitive and alone conditions.’, (Hypothesis 6) ‘Offering and asking for help will be different for male partners and female partners, in verbal, intuitive and alone conditions.’

Doing chores together with one’s spouse brings happiness [50] while doing them alone is correlated to higher levels of loneliness and intense negative feelings [51]. Moreover, for women, doing most of the housework alone impacts their perceived marriage quality and they score lower on marital closeness scales than women who share chores with their partners [52]. However, when it comes to spending time together as a couple, partners are more willing to forgo doing household chores together, rather than narrowing joint leisure time [53].

## 5. Methodology

The present article is an original research article, probably the first to investigate and compare several aspects of judgement referring to helping behaviour in a couple’s situation, by gender. The methodology is based on self-reported answers based on vignettes.

### 5.1. Participants

Children and adults were recruited for the study through classrooms or the internet, and they were forwarded to a link with a Google Forms questionnaire. The raw database contained 642 subjects, 18.1% girls (Mage = 14.6), 18.1% boys (Mage = 13.2), 17.1% male partners (Mage = 36.9), and 46.7% female partners (Mage = 33.9). Female partners had a mean age of their marriage duration of 10.02 (SD = 8.19) and male partners of 7.72 (5.76). All adult respondents were married.

Due to the main researcher’s involvement with the Faculty of Educational Sciences, which has a predominately female student body, there were more female partners who answered. The questionnaire completion time ranged from 4 to 7 min.

All children came from intact families. The children were recruited from schools and they received parental permission to fill up the questionnaires. Boys, girls, male partners, and female partners who took part in the research were not related as a family.

Married adults and teenage children were respondents in the current study. Our justification for this is as follows. The topic of the present study is new and we could not locate any previous research on it. We predicted, based on direct observations, that there will be differences between male partners’ and female partners’ judgements regarding domestic chores. We are not focused in the present study on researching whether the causes are the result of nurture or nature. However, it is assumed that children, who by the time they reach adolescence have observed their parents and have formed a personal judgement concerning couple relationships and duties and responsibilities such as household chores, are well aware of how couples live their lives and the specifics of living together as spouses [54]. Thus, we have included adolescents as representative of expectations without personal experience in a couple, versus adults who have expectations but also have a personal experience of living as a couple, regarding domestic chores, as an exploratory measure with potential implications for future research.

### 5.2. Collecting Data and Preparing Data Bases

The data were collected individually, using auto-reported questionnaires. The respondents answered the questions online in Google Forms. The questionnaire was distributed by a link to different schools or groups of individuals who corresponded to the profile needed for the study. In order to perform the analyses, the data were transformed to fit a 2 × 2 or a 2 × 3 Chi-square test. The analyses were performed using Jamovi 2.3.24 (Desktop version) [55], IBM SPSS 26 [56], and Microsoft Excel 2010 [57].

### 5.3. Strategy of Analysis

The present research calculated frequencies which were analysed using the Chi2 test of association in Jamovi, which indicated whether there are significant associations between the two categorical variables. Further on, we used a comparison test of two proportions and we obtained a Chi2 and a ***p***-value for each comparison. Thus, we tested whether there are significant differences between pairs of proportions.

### 5.4. Instruments

In order to measure how the respondents judge whether to ask or offer help, we created four vignettes, with four predefined answers and asked participants to choose one answer that best fits their course of action. We created a vignette for boys, girls, male partners, and female partners. Vignettes are scenarios in written forms, representative of specific life situations, which, in the present case, are used to clarify people’s judgements [58].

**Figure 1:**
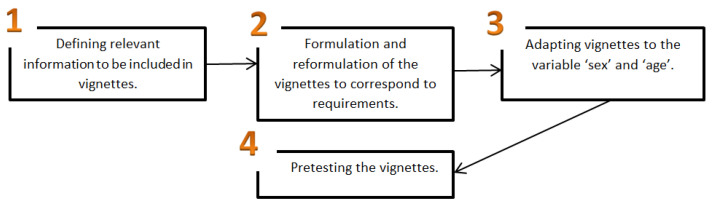
A flow chart indicating the steps for constructing the vignettes. Source: The graphic was created by the authors using Microsoft Word 2010 [57].

The process of creating vignettes included several stages and standards (see Figure 1): First, we defined the most important information a vignette must incorporate and how the vignettes can refer to the experiences the respondents have. Being ‘verbal’ was defined as referring to using words such as questions in asking or in offering help, while being ‘intuitive’ was defined as referring to doing chores without using words in offering or in asking for help. Doing chores ‘alone’ meant no need for help from the partner at all. Secondly, we formulated four short vignettes and then reformulated them until they sounded natural, represented the situation intended well, and were fluent. Adults were asked to imagine the following situation and children were asked to imagine that they are mum and dad. Then, the text was the same for all respondents only that vignettes were adapted to refer to boys or girls or male partners and female partners. The vignettes asked participants to picture in their mind that they are mum or dad (for children) and answer for themselves as if they were a mom and a dad or to imagine the following situation (for adults). One day they come home. They are tired and the house is a mess. Then, they were asked: what would they prefer to happen so that you/your female partner/your male partner can finish tidying the house? The respondents were asked to make a forced choice and circle one answer from four options: (1) You ask your male partner/female partner verbally to help you tidy the house so that he/she helps you; (2) You wait until your male partner/female partner asks you if you need help and then you ask for help and he/she helps you; (3) You expect your female partner/male partner to see that you need help with home chores, and help you without you asking verbally; (4) You do the house by yourself, without asking your female partner/male partner verbally for help. Then, we pretested the items and the respondents indicated different problems, which were corrected (formulations, clarity, length of text, better-written formulations).

In addition, information was collected regarding the sex of the children, the sex of the parents, family income, years of marriage, age of children, or the age of parents.

The vignettes and the question type-items were included in a Google Form Sheet so that respondents would answer them online.

## 6. Results

### 6.1. Descriptive Analysis

In order to analyse the raw data, we performed a frequency analysis regarding all the situations implied in the study. Table 1 indicates frequencies and percentages (in parentheses) for the type of respondents, choice of doing chores, and asking for or offering help.

In order to test the hypotheses above, we performed multiple Chi-square tests of independence, in Jamovi, to assess whether there was a significant association between variables. Different databases were created for each Chi-square test. The initial answer categories were recoded: ‘Observing without words’ became ‘intuitive’, and ‘Asking in words’ and ‘Waiting to be asked’ became ‘verbal’, while the ‘Alone’ condition remained unmodified. The results of the analyses are presented in Table 1.

### 6.2. Testing the Hypotheses

**Testing hypothesis 1:** ‘There will be differences between boys and girls regarding being intuitive, verbal or doing chores alone, in the condition of asking and offering help.’

The chi-square test of independence indicated that there was a significant association between the sex of respondents (‘boys’ and ‘girls’) and the respondents’ choice regarding chores (‘intuitive’, ‘verbal’ and ‘alone’), in the offering-help condition: [χ^2^(1, *n*= 232) = 86.43, *p* < 0.00001] (see Table 2). Comparison of two independent proportions using chi-square indicated that, in the offering condition, girls were significantly more intuitive than boys [χ^2^(1) = 4.937, *p* < 0.026, M_Diff_. = 56.9%, N = 74], and boys were significantly more verbal than girls [χ^2^(1) = 39.898, *p* < 0.0001, M_Diff_. = 51.7%, N = 144], while there was no significant difference between boys and girls doing chores alone [χ^2^(1) = 0.109, *p* < 0.7418, M_Diff_. = 5.2%, N = 14], (see Table 3).

In the asking-for-help condition, the chi-square test of independence indicated that there was a significant association between the sex of respondents (‘boys’ and ‘girls’) and the respondents’ choice regarding chores (‘intuitive’, ‘verbal’, and ‘alone’): [χ^2^(1, N = 232) = 62.5, *p* < 0.00001] (see Table 2). Comparison of two independent proportions using chi-square indicated that, in the offering condition, girls were significantly more intuitive than boys [χ^2^(1) = 12.131, *p* = 0.0005, M_Diff_. = 48.3%, N = 88], and boys were significantly more verbal than girls [χ^2^(1) = 27.364, *p* < 0.0001, M_Diff_. = 50.0%, N = 126], while there was no significant difference between boys and girls doing chores alone [χ^2^(1) = 0.017, *p* < 0.8969, M_Diff_. = 1.7%, N = 18], (see Table 3). The hypothesis is confirmed.

**Testing hypothesis 2:** ‘There will be differences between male partners and female partners regarding being intuitive, verbal or doing chores alone, in the condition of asking and offering help.’

In the offering-help condition, the chi-square test of independence indicated that there was a significant association between the sex of respondents (‘male partners’ and ‘female partners’) and the respondents’ choice regarding chores (‘intuitive’, ‘verbal’ and ‘alone’): [χ^2^(1, N = 410) = 103.29, *p* < 0.00001] (see Table 2). Comparison of two independent proportions using chi-square indicated that, in the offering condition, female partners were significantly more intuitive than male partners [χ^2^(1) = 19.625, *p* = 0.0001, M_Diff_. = 54.8%, N = 224] and male partners were significantly more verbal than female partners [χ^2^(1) = 52.04, *p* < 0.0001, M_Diff_. = 55.2%, N = 170], while there was no significant difference between boys and girls doing chores alone [χ^2^(1) = 0.001, *p* < 0.9724, M_Diff_. = 0.4%, N = 16], (see Table 3).

In the asking-for-help condition, the chi-square test of independence indicated that there was no significant association between the sex of respondents (‘male partners’ and ‘female partners’) and the respondents’ choice regarding chores (‘intuitive’, ‘verbal’, and ‘alone’): [χ^2^(1, N = 410) = 4.796, *p* < 0.0908] (see Table 2). Comparison of two independent proportions using chi-square indicated that, in the asking-for-help condition, female partners were significantly more intuitive than male partners [χ^2^(1) = 0.015, *p* = 0.9033, M_Diff_. = 1.3%, N = 56] and male partners were significantly more verbal than female partners [χ^2^(1) = 2.687, *p* = 0.1012, M_Diff_. = 11.5%, N = 234], while there was no significant difference between boys and girls doing chores alone [χ^2^(1) = 0.944, *p* < 0.3312, M_Diff_. = 10.2%, N = 120], (see Table 3). The hypothesis is partially confirmed in two out of four situations.

**Testing hypothesis 3:** ‘When offering or asking for help, girls will be similar to female partners in being intuitive, verbal, or doing the chores alone.’

In the offering-help condition, the chi-square test of independence indicated that there was no significant association between the type of respondent (‘girls’ and ‘female partners’) and the respondents’ choice regarding chores (‘intuitive’, ‘verbal’, and ‘alone’): [χ^2^(1, N = 416) = 3.673, *p* = 0.1593] (see Table 4). Comparison of two independent proportions using chi-square indicated that, in the offering condition, female partners were not significantly different in intuition as compared with girls [χ^2^(1, N = 278) = 1.913, *p* = 0.1667, M_Diff_. = 9%] and there was no significant difference between female partners and girls regarding being verbal [χ^2^(1, N = 122) = 1.175, *p* = 0.2785, M_Diff_. = 9.5%]. There was also no significant difference between female partners and girls doing chores alone [χ^2^(1, N = 16) = 0.002, *p* < 0.9653, M_Diff_. = 0.5%], (see Table 5).

In the asking-for-help condition, the chi-square test of independence indicated that there was a significant association between the type of respondent (‘girls’ and ‘female partners’) and the respondents’ choice regarding chores (‘intuitive’, ‘verbal’ and ‘alone’): [χ^2^(1, N = 416) = 99.30, *p* < 0.00001] (see Table 4). Comparison of two independent proportions using chi-square indicated that, in the asking-for-help condition, female partners were less intuitive than girls [χ^2^(1, N = 114) = 24.549, *p* < 0.0001, M_Diff_. = 48%] and girls were significantly less verbal than female partners [χ^2^(1, N = 196) = 6.823, *p* = 0.0090, M_Diff_. = 24.7%]. There was also no significant difference between female partners and girls doing chores alone [χ^2^(1, N = 106) = 2.344, *p* = 0,1257, M_Diff_. = 23.38%,], (see Table 5). The hypothesis is partially confirmed, in two out of four situations, only in the asking-for-help condition, but not in the offering-help condition.

**Testing hypothesis 4:** ‘When offering or asking for help, boys will be similar to male partners in being intuitive, verbal, or doing the chores alone.’

In the offering-help condition, the chi-square test of independence indicated that there was a significant association between the type of respondent (‘boys and ‘male partners’) and the respondents’ choice regarding chores (‘intuitive’, ‘verbal’ and ‘alone’): [χ^2^(1, N = 226) = 10.36, *p* = 0.005] (see Table 4). Comparison of two independent proportions using chi-square indicated that, in the offering condition, boys were not significantly different in intuition as compared with male partners [χ^2^(1, N = 20) = 0.345, *p* = 0.5568, M_Diff_. = 11.1%] and there was no significant difference between boys and male partners regarding being verbal [χ^2^(1, N = 192) = 1.391, *p* = 0.2382, M_Diff_. = 6.1%]. There was also no significant difference between boys and male partners doing chores alone [χ^2^(1, N = 14) = 0.100, *p* = 0.7523, M_Diff_. = 5.0%], (see Table 5).

In the asking-for-help condition, the chi-square test of independence indicated that there was a significant association between the type of respondent (‘boys’ and ‘male partners’) and the respondents’ choice regarding chores (‘intuitive’, ‘verbal’ and ‘alone’): [χ^2^(1, N = 194) = 10.420, *p* < 0.0054] (see Table 4). Comparison of two independent proportions using chi-square indicated that, in the asking-for-help condition, boys were as intuitive as male partners [χ^2^(1, N = 30) = 0.006, *p* = 0.9368, M_Diff_. = 1%] and boys were significantly more verbal than male partners [χ^2^(1, N = 164) = 3.901, *p* = 0.0483, M_Diff_. = 13.8%]. There was also no significant difference between male partners and boys doing chores alone [χ^2^(1, N = 32) = 0.871, *p* = 0,3506, M_Diff_. = 14.9%,], (see Table 5). The hypothesis is partially confirmed, in one out of four situations.

**Testing hypothesis 5:** ‘Offering and asking for help will be different for boys and girls, in verbal, intuitive, and alone conditions.’

In the intuitive condition, the chi-square test of independence indicated that there was a significant association between the type of help (‘asking’ and ‘offering’) and the sex of the respondent (‘boy’ and ‘girl’): [χ^2^(1, N = 162) = 4.940, *p* = 0.0262] (see Table 6). Comparison of two independent proportions using chi-square indicated that, in the intuitive condition, boys were not significantly different when asking, when compared with offering [χ^2^(1, N = 20) = 0.376, *p* = 0.5397, M_Diff_. = 12.77%]. There was no significant difference between asking and offering, regarding being intuitive, in the case of girls either: [χ^2^(1, N = 142) = 5.488, *p* = 0.0191, M_Diff_. = 12.77%], (see Table 7).

In the verbal condition, the chi-square test of independence indicated that there was no significant association between the type of help (‘asking’ and ‘offering’) and the sex of the respondent (‘boy’ and ‘girl’): [χ^2^(1, N = 270) = 0.1583, *p* = 0.6907] (see Table 6). Comparison of two independent proportions using chi-square indicated that, in the verbal condition, boys were not significantly different when asking, when compared to offering [χ^2^(1, N = 194) = 0.114, *p* = 07355, M_Diff_. = 2.19%]. There was no significant difference between asking and offering, regarding being verbal, in the case of girls either: [χ^2^(1, N = 76) = 0.044, *p* = 0.8340, M_Diff_. = 2.19%], (see Table 7).

In the alone condition, the chi-square test of independence indicated that there was no significant association between the type of help (‘asking’ and ‘offering’) and the sex of the respondent (‘boy’ and ‘girl’): [χ^2^(1, N = 32) = 1.362, *p* = 0.2430] (see Table 6). Comparison of two independent proportions using chi-square indicated that, in the ‘alone’ condition, boys were not significantly different when asking, as compared to offering [χ^2^(1, N = 18) = 1.268, *p* = 0.2601, M_Diff_. = 26.99%]. There was no significant difference between asking and offering, regarding doing chores alone, in the case of girls either: [χ^2^(1, N = 14) = 0.774, *p* = 0.3788, M_Diff_. = 26.99%], (see Table 7).

The hypothesis is partially confirmed, in one out of six situations.

**Testing hypothesis 6:** ‘Offering and asking for help will be different for the male partner and female partner, in verbal, intuitive, and alone conditions.’

In the intuitive condition, the chi-square test of independence indicated that there was a significant association between the type of help (‘asking’ and ‘offering’) and the sex of the respondent (‘husband’ and ‘wife’): [χ^2^(1, N = 280) = 14.933, *p* = 0.0001] (see Table 6). Comparison of two independent proportions using chi-square indicated that, in the intuitive condition, in the case of male partners, there were no significant differences between asking and offering [χ^2^(1, N = 30) = 1.760, *p* = 0.1846, M_Diff_. = 17.86%]. There was a significant difference between asking and offering, regarding being intuitive, in the case of female partners: [χ^2^(1, N = 250) = 12.184, *p* = 0.0005, M_Diff_. = 17.86%], (see Table 7). In the intuitive condition, m offered and asked equally, while female partners offered significantly more than they asked.

In the verbal condition, the chi-square test of independence indicated that there was a significant association between the type of help (‘asking’ and ‘offering’) and the sex of the respondent (‘male partner’ and ‘female partner’): [χ^2^(1, N = 404) = 20.152, *p* = 0.00001] (see Table 6). Comparison of two independent proportions using chi-square indicated that, in the verbal condition, in the case of the male partner there were significant differences between asking and offering [χ^2^(1, N = 162) = 7.968, *p* = 0.0048, M_Diff_. = 22.17%]. There was a significant difference between asking and offering, regarding being verbal, in the case of female partners: [χ^2^(1, N = 242) = 11.115, *p* = 0.0009, M_Diff_. = 22.17%], (see Table 7). These results indicate that in the intuitive condition, male partners offer significantly more often than they ask for help, and female partners ask more than they offer.

In the alone condition, the chi-square test of independence indicated that there was no significant association between the type of help (‘asking’ and ‘offering’) and the sex of the respondent (‘male partner’ and ‘female partner’): [χ^2^(1, N = 270) = 0.018, *p* = 0.8922] (see Table 6). Comparison of two independent proportions using chi-square indicated that, in the ‘alone’ condition, male partners were not significantly different when asking, as compared with offering [χ^2^(1, N = 28) = 0.050, *p* = 0.2601, M_Diff_. = 5%]. There was no significant difference between asking and offering, regarding doing chores alone, in the case of female partners either: [χ^2^(1, N = 108) = 0.162, *p* = 0.6875, M_Diff_. = 5%], (see Table 7).

The hypothesis is partially confirmed, in three out of six situations.

## 7. Discussions

The purpose of the present research was to investigate judgement differences in males and females (married and unmarried) regarding the offering and asking for help in a very common couple situation, that of domestic chores.

Firstly, results indicated that in the offering condition girls are more intuitive than boys and boys are more verbal than girls, while there is no difference between them in doing chores alone. In addition, in the offering condition, female partners are more intuitive than male partners and male partners are more verbal than female partners, while there is no difference between them in doing chores alone. In the asking-for-help condition, girls remain again more intuitive than boys, while boys remain more verbal than girls. The difference is that, in the asking condition, there is no difference between male partners and female partners either in the intuitive condition or in the verbal condition. As expected, in general, the research literature confirms that men are more verbal and women more intuitive [9,13]. A previous study [62] found that women prefer using face-saving strategies laterally, with people with power similar to theirs. The study suggests that in close relationships women might choose indirect/intuitive help-seeking strategies. However, this study was performed in an organisational setting, which raises the question of applicability to the marriage setting.

It is very likely that men are typically more direct in initiating helping behaviour and women more indirect (using words but not mentioning help)/intuitive (no words) owing to social learning factors. Johnson [63] states that women are expected to use indirect/intuitive styles as they might be seen as less feminine otherwise. Men might be more verbal (direct) than intuitive (indirect) because they are more powerful. Studies show that men usually have more power than women so they are consequently more verbal than intuitive as compared to women [64,65]. Men expect compliance so they use direct strategies such as verbal, while women are less likely to expect compliance so they use indirect strategies (using words but not requesting or offering help, or intuitive strategies, meaning no words at all) [66]. Sex differences in communication may be an effect of power [3].

Secondly, our analyses indicated that in general there are no differences between the same-sex respondents except for three, all in the asking-for-help condition. In this condition, male partners are less verbal than boys, while female partners are less intuitive than girls and more verbal than girls. As boys’ verbal level remains high, and girls’ intuitive level remains high and their verbal level remains low, these results seem to indicate that female partners’ judgement compromises more in favour of their male partners when asking for help than girls’ judgement who are not in a relationship. Female partners are more verbal and less intuitive than girls when asking for help. In addition, these results indicate that male partners’ judgement compromises in favour of their female partners, when offering help, thus they become less verbal than boys, whose judgement is more verbal than that of male partners. The results indicate that female partners compromise more in favour of their male partners (intuitive and verbal) and male partners compromise less (only verbal) than boys. Male partners and female partners seem to approach the opposite sex dominantly, as found in hypotheses one and two. The couple’s relationship might be a reason to compromise and maintain communication and closeness. The literature indicates that ‘girls are often more motivated biologically than men to please parents, teachers, and peers as they strive to establish and maintain relationships’, [67], (p. 23). This is because of high levels of oxytocin (often referred to as a ‘tend and befriend hormone’). The same author affirms that men are more inclined to be competitive and self-reliant.

Thirdly, girls seem to offer intuitively more than asking for help intuitively, while the same seems to maintain for female partners. Female partners offer intuitively more than expecting without words (‘ask’ and ‘intuitive’ conditions). Boys and male partners do not report any differences between asking for help and offering help intuitively.

There is no difference between asking and offering for help verbally, in the case of boys or girls. However, in the case of male partners and female partners, male partners offer verbally more, and ask for help verbally less, while female partners offer verbally less, and ask verbally more. These changes can be attributed to the partners’ adaptation process of living together as a couple.

No differences were reported between doing the chores alone, in the case of boys, girls, male partners, and female partners.

## 8. Conclusions

Generally, research in the field has focused on finding gender differences and understanding whether they are produced by nature or nurture. However, in the couple’s relationship, it seems more important to find gender differences and to understand how they disconnect or connect partners in a couple. Gender differences in judgements are often seen as less important than genetic gender differences. Yet, cultural or judgement gender differences might be as important as genetically based differences since they impact the couple-relationship quality and contribute to a happy or disastrous intimate relationship. The simple existence of gender differences implies a different perspective on reality and a need to make an effort to understand a different perspective and to adapt to it, for the sake of a functional couple relationship. Gender seems to be a salient variable in understanding life as a couple [29]. Yet, more studies are necessary in order to understand how much the present findings can be generalized.

Our study has some limits and strong points too. Our research is based on frequencies and proportions and other studies might continue to document the issue of domestic chores in couple relationships. The data is self-reported, and other research designs might contribute to the research topic.

This paper offers many opportunities for further research as it triggers several research challenges. Firstly, further research should investigate couple interactions in very specific settings, which are relevant to intimate relationship life quality. Too often, research focused on general differences between males and females, ignoring specific differences in couple relationships. Secondly, future studies should add a new variable, and compare respondents’ ideal expectations versus their actual reality expectations, concerning their answers to the questions. We speculate that there might be significant differences between male and female respondents concerning the role of intuitive or verbal preference. Female partners, for example, might declare that they expect men to be intuitive, but they become verbal for the sake of a functional couple relationship. In addition, although we could not find many significant differences in the condition ‘alone’ between males and females, other variables might influence the choice of doing chores alone. Uninvolved male partners, for example, might lead female partners to the decision to choose to do chores alone, as they become angry or are faced with a no-choice situation and must do chores by themselves. In addition, other variables such as marital satisfaction, gender norms or gender identification could bring even more light to the present topic. Thirdly, future research should investigate how the way in which help is requested and offered conveys love messages within the couple and how words versus intuitive approaches convey closeness in married life. Fourthly, research should confirm our research by investigating more thoroughly whether female partners compromise more and male partners less, in couple relationships. Finally, our results could be confirmed by longitudinal or experimental studies which would probably bring more light to the issue of domestic chores.

## 9. Implications

The present research started from observations in couple therapy practice and the results confirm that there are differences regarding how females and males perceive offering and asking for help. Males prefer the verbal way of initiating help and females prefer the intuitive way of initiating help with chores, probably as a proof of affection within the couple.

Our research might have some practical implications. Scientifically, our research indicates that these differences might be important as long as they are related to a very frequent occupation in the life of a couple—domestic chores. This single piece of research might become a starting point for other studies, which could investigate being intuitive or verbal in helping behaviour in couple relationships, and in other families of couple situations. In addition, our research raises the question of how much verbal communication and how much intuition is necessary in a couple’s life, as the classical couple paradigm suggests that couples must verbally communicate, in order to have a functional relationship. Yet, this research indicates that words are not always necessary. Educationally, intuition can be ‘deliberately cultivated through parental practices’ [68] (p. 72), which means that courses could teach males specifically to become more intuitive and females to speak about their needs more regarding chores or similar needs. If couple partners need to learn to be intuitive, then this must become a to-do issue in couple education programs. Practically, couples can become more functional if they understand the particular differences between males and females, as our findings underline that in couple relationships being intuitive might represent an important ability.

## Figures and Tables

**Table 1:** Responses of boys, girls, male partners, and female partners in the following conditions: alone, observing without words, asking in words, or waiting to be asked.

		Alone *n* (%)	Observing without Words *n* (%)	Asking in Words *n* (%)	Waiting to Be Asked *n* (%)
Boys	Asking for help, n = 116	8 (7)	16 (14)	44 (38)	48 (41)
	Offering help, n = 116	10 (9)	4 (3)	26 (22)	76 (66)
Girls	Asking for help, n = 116	10 (9)	72 (62)	24 (21)	10 (9)
	Offering help, n = 115	4 (3)	70 (61)	36 (30)	6 (5)
Male partners	Asking for help, n = 110	24 (22)	14 (13)	42 (38)	30 (27)
	Offering help, n = 110	4 (4)	16 (15)	44 (40)	46 (42)
Female partners	Asking for help, n = 300	96 (32)	42 (14)	148 (49)	14 (5)
	Offering for help, n = 300	12 (4)	208 (69)	56 (19)	24 (8)

Source: The graphic was created by the authors using Microsoft Word 2010 [57].

**Table 2:** Comparisons of females and males in the intuitive and verbal condition of asking for or offering help.

Respondents	Intuitive *n*/Expected	Verbal *n*/Expected	Alone *n*/Expected	Total *n*	Chi-Square Tests of Independence
***Offering help***					
Boy	4 (37)	102 (72)	10 (7)	116	χ^2^(1) = 86.43 *p* < 0.00001 n = 232
Girl	70 (37)	42 (72)	4 (7)	116	
Total	74	144	14	232	
Male partner	16 (60.10)	90 (45.61)	4 (4.29)	110	χ^2^(1) = 103.29 *p* < 0.00001 n = 410
Female partner	208 (163.90)	80 (124.39)	12 (11.71)	300	
Total	224	170	16	410	
** Asking for help**					
Boy	16 (44)	92 (63)	8 (9)	116	χ^2^(1) = 62.5 *p* < 0.00001 n = 232
Girl	72 (44)	34 (63)	10 (9)	116	
Total	88	126	18	232	
Male partner	14 (15.02)	72 (62.78)	24 (32.20)	110	χ^2^(1) = 4.796 *p* = 0.0908 n = 410
Female partner	42 (40.98)	162 (171.22)	96 (87.80)	300	
Total	56	234	120	410	

Source: The graphic was created by the authors using Microsoft Word 2010 [57].

**Table 3:** Comparisons of females and males in asking or offering help in intuitive, verbal, and alone conditions.

Respondents	Intuitive *n*/(%)	Verbal *n*/(%)	Alone *n*/(%)	Total *n*/(%)
Offering help				
Boy	4 (3.4)	102 (87.9)	10 (8.6)	116 (100)
Girl	70 (60.3)	42 (36.2)	4 (3.4)	116 (100)
Comparing two proportion test	χ^2^(1) = 4.937 *p* = 0.026 ^a^, M_Diff_. = 56.9	χ^2^(1) = 39.898, p < 0.0001 ^a^, M_Diff_. = 51.7	χ^2^(1) = 0.109, *p* = 0.7418 ^a^, M_Diff_. = 5.2	
Total	74	144	14	232 (100)
Male partner	16 (14.5)	90 (81.9)	4 (3.6)	110 (100)
Female partner	208 (69.3)	80 (26.7)	12 (4.0)	300 (100)
Comparing two proportion test	χ^2^(1) = 19.625, p < 0.0001 ^a^, M_Diff_. = 54.8	χ^2^(1) = 52,04 p < 0.0001 ^a^, M_Diff_. = 55.2	χ^2^(1) = 0.001, *p* = 0.9724 ^a^, M_Diff_. = 0.4	
Total	224	170	16	410 (100)
Asking for help				
Boy	16 (13.8)	92 (79.3)	8 (6.9)	116 (100)
Girl	72 (62.1)	34 (29.3)	10 (8.6)	116 (100)
Comparing two proportion test	χ^2^(1) = 12.130, *p* = 0.0005 ^a^, M_Diff_. = 48.3	χ^2^(1) = 27.364, p < 0.0001 ^a^, M_Diff_. = 50.0	χ^2^(1) = 0.017 *p* = 0.8969 ^a^, M_Diff_. = 1.7	
Total	88	126	18	232 (100)
Male partner	14 (12.7)	72 (65.5)	24 (21.8)	110 (100)
Female partner	42 (14)	162 (54)	96 (32)	300 (100)
Comparing two proportion test	χ^2^(1) = 0.015, *p* = 0.9033 ^a^, M_Diff_. = 1.3	χ^2^(1) = 2.687, *p* = 0.1012 ^a^, M_Diff_. = 11.5	χ^2^(1) = 0.944, *p* = 0,3312 ^a^, M_Diff_. = 10.2	
Total	56	234	120	410 (100)

Source: The graphic was created by the authors using Microsoft Word 2010 [57]. Note: ^a^ Comparison of proportions according to Campbell [59], Richardson [60], and Altman et al. [61].

**Table 4:** Comparisons of the same-sex respondents by intuitive and verbal means in the condition of offering and asking for help.

Respondents	Intuitive n	Verbal *n*	Alone	Total *n*	Chi-Square Tests of Independence
*Offering-help*					
Boy	4 (10.27)	102 (98.55)	10 (7.19)	116	χ^2^(1) = 10.36 *p* = 0.005 n = 226
Male partner	16 (9.73)	90 (93.45)	4 (6.81)	110	
*Total*	20	192	14	226	
Girl	70 (77.52)	42 (34.02)	4 (4.46)	116	χ^2^(1) = 3.673 *p* = 0.1593 n = 416
Female partner	208 (200.48)	80 (87.98)	12 (11.54)	300	
*Total*	*278*	*122*	*16*	*416*	
*Asking-for-help*					
Boy	16 (15.40)	92 (84.18)	8 (16.42)	116	χ^2^(1) = 10.420 *p* = 0.0054 n = 194
Male partner	14 (14.60)	72 (79.82)	24 (15.58)	110	
*Total*	30	164	32	226	
Girl	72 (31.79)	34 (54.65)	10 (29.56)	116	χ^2^(1) = 99.30 *p* < 0.00001 n = 416
Female partner	42 (82.21)	162 (141.35)	96 (76.44)	300	
*Total*	114	196	106	416	

Source: The graphic was created by the authors using Microsoft Word 2010 [57].

**Table 5:** Comparisons of the same-sex respondents by intuitive and verbal means in the condition of offering and asking for help.

Respondents	Intuitive *n* (%)	Verbal *n* (%)	Alone (%)	Total *n*
Offering-help				
Boy	4 (3.5)	102 (87.9)	10 (8.6)	116 (100)
Male partner	16 (14.6)	90 (81.8)	4 (3.6)	110 (100)
Comparing two proportion test	χ^2^(1) = 0.345, *p* = 0.5568 ^a^, Diff. = −11.1	χ^2^(1) = 1.391, *p* = 0.2382 ^a^, Diff. = 6.1	χ^2^(1) = 0.100, *p* = 0.7523 ^a^, Diff. = 5.0	
Total	20	192	14	226 (100)
Girl	70 (60.3)	42 (36.2)	4 (3.5)	116 (100)
Female partner	208 (69.3)	80 (26.7)	12 (4.0)	300 (100)
Comparing two proportion test	χ^2^(1) = 1.913, *p* = 0.1667 ^a^, Diff. = 9.0	χ^2^(1) = 1.175, *p* = 0.2785 ^a^, Diff. = 9.5	χ^2^(1) = 0.002, *p* = 0.9653 ^a^, Diff. = 0.5	
Total	278	122	16	416 (100)
Asking-for-help				
Boy	16 (13.7)	92 (79.3)	8 (6.90)	116 (100)
Male partner	14 (12.7)	72 (65.5)	24 (21.8)	110 (100)
Comparing two proportion test	χ^2^(1) = 0.006, *p* = 0.9368 ^a^, Diff. = 1	χ^2^(1) = 3.901, *p* = 0.0483 ^a^, Diff. = 13.8	χ^2^(1) = 0.871, *p* = 0.3506 ^a^, Diff. = 14.9	
Total	30	164	32	226 (100)
Girl	72 (62.0)	34 (29.3)	10 (8.62)	116 (100)
Female partner	42 (14)	162 (54)	96 (32)	300 (100)
Comparing two proportion test	χ^2^(1) = 24.549, p < 0.0001 ^a^, Diff. = 48	χ^2^(1) = 6.823, *p* = 0.0090 ^a^, Diff. = 24.7	χ^2^(1) = 2.344, *p* = 0.1257 ^a^, Diff. = 23.38	
Total	114	196	106	416 (100)

Source: The graphic was created by the authors using Microsoft Word 2010 [57]. Note: ^a^ Comparison of proportions according to Campbell [60], Richardson [61], and Altman et al. [61].

**Table 6:** Comparisons of offering and asking for help situations between males and females, in intuitive or verbal conditions.

Respondents	Boy *n*/(Expected)	Girl *n*/(Expected)	Total n/(Expected)	Chi-Square Tests of Independence
Intuitive				χ^2^ (1) = 4,940 ^a^ *p* = 0.0262 n = 162
Asking	16 (10.86)	72 (77.14)	88	
Offering	4 (9.14)	70 (64.86)	74	
Total n	20	142	162	
Verbal				χ^2^ (1) = 0.1583 *p* < 0.6907 n = 270
Asking	92 (90.53)	34 (35.47)	126	
Offering	102 (103.47)	42 (40.53)	144	
Total n	194	76	270	
Alone				χ^2^ (1) = 1.362 ^a^ *p* = 0.2430 n = 32
Asking	8 (10.12)	10 (7.88)	18	
Offering	10 (7.88)	4 (6.12)	14	
Total n	18	14	32	
**Respondents**	**Male Partner** ***n*/(Expected)**	**Female Partner** ***n*/(Expected)**	**Total**	**Chi-Square Tests** **of Independence**
Intuitive				χ^2^ (1) = 14.933 *p* < 0.0001 n = 280
Asking	14 (6)	42 (50)	56	
Offering	16 (24)	208 (200)	224	
Total n	30	250	280	
Verbal				
Asking	72 (93.83)	162 (140.17)	234	χ^2^ (1) = 20.152 *p* < 0.00001 n = 404
Offering	90 (68.17)	80 (101.83)	170	
Total n	162	242	404	
Alone				χ^2^ (1) = 0.018 ^a^ *p* < 0.8922 n = 270
Asking	24 (24.71)	96 (95.29)	*120*	
Offering	4 (3.29)	12 (12.71)	*16*	
*Total n*	*28*	*108*	*136*	

Source: The graphic was created by the authors using Microsoft Word 2010 [57]. Note: ^a^ Chi-square with Yates correction as one group has a value under 10 frequencies.

**Table 7:** Comparisons of offering and asking for help situations between males and females, in intuitive or verbal conditions.

Respondents	Boy *n*/(%)	Girl *n*/(%)	Total *n*/(%)
Intuitive			
Asking	16 (18.18)	72 (81.82)	88 (100)
Offering	4 (5.41)	70 (94.59)	74 (100)
	χ^2^(1) = 0.376, *p* = 0.5397 ^a^, M_Diff_. = 12.77	χ^2^(1) = 5.488, *p* = 0.0191 ^a^, M_Diff_. = −12.77	
Total n	20	142	162 (100)
Verbal			
Asking	92 (73.02)	34 (26.98)	126 (100)
Offering	102 (70.83)	42 (29.17)	144 (100)
	χ^2^(1) = 0.114, *p* = 0.7355 ^a^, M_Diff_. = 2.19	χ^2^(1) = 0.044, *p* = 0.8340 ^a^, M_Diff_. = −2.19	
Total n	194	76	270 (100)
Alone			
Asking	8 (44.44)	10 (55.56)	18 (100)
Offering	10 (71.43)	4 (28.57)	14 (100)
	χ^2^(1) = 1.268, *p* = 0.2601 ^a^, M_Diff_. = −26.99	χ^2^(1) = 0.774, *p* = 0.3788 ^a^, M_Diff_. = 26.99	
Total n	18	14	32 (100)
Respondents	Male partner n/(%)	Female partner n/(%)	Total n/(%)
Intuitive			
Asking	14 (25)	42 (75)	56 (100)
Offering	16 (7.14)	208 (92.86)	224 (100)
	χ^2^(1) = 1.760, *p* = 0.1846 ^a^, M_Diff_. = 17.86	χ^2^(1) = 12.184, *p* = 0.0005 ^a^, M_Diff_. = −17.86	
Total n	30	250	280 (100)
Verbal			
Asking	72 (30.77)	162 (69.23)	234 (100)
Offering	90 (52.94)	80 (47.06)	170 (100)
	χ^2^(1) = 7.968, *p* = 0.0048 ^a^, M_Diff_. = −22.17	χ^2^(1) = 11.115, *p* = 0.0009 ^a^, M_Diff_. = 22.17	
Total n	162	242	404 (100)
Alone			
Asking	24 (20)	96 (80)	120 (100)
Offering	4 (25)	12 (75)	16 (100)
	χ^2^(1) = 0.050, *p* = 0.8225 ^a^, M_Diff_. = 5	χ^2^(1) = 0.162, *p* = 0.6875 ^a^, M_Diff_. = 5	
Total n	28	108	136 (100)

Source: The graphic was created by the authors using Microsoft Word 2010 [57]. Note: ^a^ Comparison of proportions according to Campbell [59], Richardson [60], and Altman et al. [61].

## Data Availability

Not applicable.
